# Terpenoids from *Chloranthus elatior*

**DOI:** 10.1007/s13659-012-0039-7

**Published:** 2012-07-02

**Authors:** Chang-Li Sun, Huan Yan, Xu-Hong Li, Xue-Fang Zheng, Hai-Yang Liu

**Affiliations:** 1 State Key Laboratory of Phytochemistry and Plant Resources in West China, Kunming Institute of Botany, Chinese Academy of Sciences, Kunming, 650201 China; 2 College of Forestry, Southwest Forestry University, Kunming, 650224 China

**Keywords:** *Chloranthus elatior*, Chloranthaceae, sesquiterpenoid, chlorelactone, elatiorlabdane

## Abstract

Two new eudesmane-type sesquiterpenoid lactones, named chlorelactone A (**1**) and chlorelactone B (**2**), and one new labdane-type diterpenoid, named elatiorlabdane (**3**), along with seven known sesquiterpenoids and one known disesquiterpenoid were isolated from the whole plants of *Chloranthus elatior*. Their structures and relative configurations were established on the basis of extensive spectroscopic analysis.

**Electronic Supplementary Material:**

Supplementary material is available for this article at 10.1007/s13659-012-0039-7 and is accessible for authorized users.

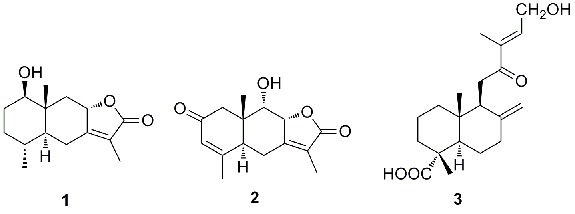

## Introduction

The genus *Chloranthus* (Chloranthaceae) has about 17 species in the world, and 13 species and 5 varieties of this genus are distributed in China.[1,2] Some plants of this genus have been used in Chinese folk medicine for the treatment of bone fractures.[3] Previous work on this genus indicated that sesquiterpenoids and disesquiterpenoids were their major secondary metabolites.[4–11] Disesquiterpenoids were reported to exhibit inhibition of cell adhesion molecule expression, antifungal activity, anti-HIV-1 activity, tumor growth inhibitory activity, potent and selective inhibition on the delayed rectifier ($I_k$) $K^+$ current.[10,11] The tyrosinase inhibitory activity and cytotoxicity of sesquiterpenoids also have been reported.[12,13]

*Chloranthus elatior* is a perennial plant and grows in the Southwest of China. Recent phytochemical investigation on this species reported 18 compounds including nine sesquiterpenoids and four diterpenoids.[8] In the course of our group searching for new sesquiterpenoids from *Chloranthus* species, two new eudesmane-type sesquiterpenoid lactones, named chlorelactone A (**1**) and chlorelactone B (**2**), and one new labdane-type diterpenoid, named elatiorlabdane (**3**), along with seven known sesquiterpenoids, spathulenol (**4**),[14] 4*α*-hydroxy-5*α*,8*β*(H)-eudesm-7(11)-en-8,12-olide (**5**),[6] 4*α*-hydroxy-5*α*,8*α*(H)-eudesm-7(11)-en-8,12-olide (**6**),[6] neolitacumone B (**7**),[15] chlorantholide D (**8**),[8] 4*α*,8*β*-dihydroxy-5*α*(H)-eudesm-7(11)-en-8,12-olide (**9**),[6] chloranthalic acid (**10**),[16] and one disesquiterpenoid, chloramultilide C (**11**)[17] were isolated from the whole plants of *C. elatior*. Herein, we report the isolation and structural elucidation of these compounds.

## Results and Discussion

Chlorelactone A (**1**) was obtained as a white amorphous powder, and its molecular formula was established as $C_{15}H_{22}O_3$ by the postive HRESIMS at *m/z* 273.1463 [M + Na]$^+$ (calcd. 273.1466). The $^1$H NMR spectrum (Table 1) showed the presence of two tertiary methyl singlets at $\delta_H$ 1.00 and 1.82, a secondary methyl at $\delta_H$ 0.93 (d, $J$ = 6.0 Hz), and two

*To whom correspondence should be addressed. E-mail: haiyangliu@mail.kib.ac.cn

**Table 1.** $^{1}$**H NMR and** $^{13}$**C NMR spectroscopic data of compounds 1 and 2 (*δ* in ppm)**

| | 1[a] | | 2[b] | |
|---|---|---|---|---|
| pos. | $\delta_H$ (*J* in Hz) | $\delta_C$ | $\delta_H$ (*J* in Hz) | $\delta_C$ |
| 1α | 3.26 (dd, 11.4, 4.1) | 78.2 (d) | 2.88 (d, 16.4) | 49.5 (t) |
| 1β | | | 2.09 (d, 16.4) | |
| 2α | 1.62 (m) | 30.1 (t) | | 201.9 (s) |
| 2β | 1.74 (m) | | | |
| 3α | 1.75 (m) | 33.8 (t) | 5.89 (s) | 126.8 (d) |
| 3β | 1.06 (m) | | | |
| 4 | 1.48 (m) | 31.6 (d) | | 164.7 (s) |
| 5 | 0.86 (m) | 50.6 (d) | 2.96 (dd, 13.0, 0.9) | 41.9 (d) |
| 6α | 2.85 (dd, 14.1, 4.0) | 26.3 (t) | 3.16 (dd, 13.7, 13.0) | 25.6 (t) |
| 6β | 2.02 (dd, 14.1, 13.2) | | 2.44 (dd, 13.7, 0.9) | |
| 7 | | 162.4 (s) | | 160.0 (s) |
| 8 | 4.81 (dd, 11.0, 6.4) | 78.4 (d) | 5.18 (br. s) | 81.1 (d) |
| 9α | 0.93 (dd, 12.3, 11.0) | 44.5 (t) | 3.84 (br. s) | 75.0 (d) |
| 9β | 2.76 (dd, 12.3, 6.4) | | | |
| 10 | | 40.3 (s) | | 44.7 (s) |
| 11 | | 120.1 (s) | | 123.7 (s) |
| 12 | | 175.2 (s) | | 177.6 (s) |
| 13 | 1.82 (s) | 8.5 (q) | 1.86 (s) | 8.2 (q) |
| 14 | 1.00 (s) | 11.4 (q) | 1.08 (s) | 16.2 (q) |
| 15 | 0.93 (d, 6.0) | 19.8 (q) | 2.07 (s) | 22.2 (q) |

[a]Run in $CDCl_3$ and 600 MHZ; [b]Run in $CD_3OD$ and 400MHZ

oxymethine protons at $\delta_H$ 3.26 (dd, *J* = 11.4, 4.1 Hz) and 4.81 (dd, *J* = 11.0, 6.4 Hz). The $^{13}$C NMR spectrum (Table 1) displayed 15 carbon signals, including an *α*,*β*-unsaturated ester carbonyl at $\delta_C$ 175.2 (s) and two olefinic carbons at $\delta_C$ 120.1 (s) and 162.4 (s). The above NMR spectroscopic data suggested that **1** was an eudesmane-type sesquiterpenoid lactone, which has been widely discovered from this genus.[4–8] Comparison of NMR data of **1** with those of neolitacumone B (**7**)[15] indicated that **1** differed from **7** by the presence of a methyl ($\delta_H$ 0.93, d, *J* = 6.0 Hz and $\delta_C$ 19.8) and a methine ($\delta_H$ 1.48, m and $\delta_C$ 31.6) instead of one terminal double bond in **7**, which was reasonable to assume that **1** is the 4,15-dihydrogenation derivative of **7**. In the HMBC spectrum (Figure 1), the correlations of Me-15 ($\delta_H$ 0.93) with C-3 ($\delta_C$ 33.8), C-4 ($\delta_C$ 31.6), and C-5 ($\delta_C$ 50.6) were observed, which confirmed the above assumption. In addition, the HMBC correlations from $\delta_H$ 1.00 (Me-14) to $\delta_C$ 78.2 (C-1), $\delta_C$ 50.6 (C-5), $\delta_C$ 44.5 (C-9), and $\delta_C$ 40.3 (C-10) hinted that one hydroxyl group was attached at C-1. The relative configuration of **1** was established by a ROESY experiment (Figure 2), in which the correlations from Me-14 to H-4 and H-8 indicated that they were on the same side of the rings and were randomly assigned being *β*-oriented. Consequently, the ROESY correlations of Me-15/H-5 and H-5/H-1 suggested that H-1 and H-5 had *α*-orientation. Thus, compound **1** was identified as 1*β*-hydroxy-4*β*,5*α*,8*β*(H)-eudesm-7(11)-en-12,8*α*-olide.

**1** **2** **3**

**Figure 1.** $^{1}$H-$^{1}$H COSY ( ▬ ) and key HMBC (→) correlations of **1**–**3**

Chlorelactone B (**2**) had a molecular formula of $C_{15}H_{18}O_4$ based on the HREIMS at *m/z* 262.1211 $[M]^+$ (calcd. 262.1205). The NMR data of **2** (Table 1) were very similar to those of chlorantholide B[8] except for the presence of one more oxymethine ($\delta_H$ 3.84, br. s and $\delta_C$ 75.0) and the absence of a methylene. The oxymethine was assigned at C-9 according to the $^{1}$H-$^{1}$H COSY correlations of $\delta_H$ 5.18 (H-8) with $\delta_H$ 3.84 (H-9) and HMBC correlations (Figure 1) from $\delta_H$ 1.08 (Me-14) to $\delta_C$ 49.5 (C-1), $\delta_C$ 41.9 (C-5), $\delta_C$ 75.0 (C-9), and $\delta_C$ 44.7 (C-10). The ROESY (Figure 2) correlations from $\delta_H$ 1.08 (Me-14) to $\delta_H$ 5.18 (H-8) and 3.84 (H-9) indicated that H-8 and H-9 were cofacial and were arbitrarily assigned with a *β*-orientation. Accordingly, the structure of **2** was elucidated as 9*α*-hydroxy-5*α*,8*β*(H)-2-oxoeudesma-3,7(11)-dien-12,8*α*-olide.

Elatiorlabdane (**3**), white amorphous powder, was assigned as $C_{20}H_{30}O_4$ by its exact mass at *m/z* 334.2144 in HREIMS. The IR spectrum showed hydroxyl (3442 $cm^{-1}$) and two carbonyl (1694 and 1680 $cm^{-1}$) absorptions. The UV absorptions at 228 nm indicated the presence of an *α*,*β*-unsaturated ketone. The $^{1}$H NMR spectrum (Table 2) showed three methyl proton signals at $\delta_H$ 0.80, 1.14, and 1.72, three olefinic proton resonances at $\delta_H$ 4.32 (1H, s, H-17), 4.68 (1H, s, H-17), and 6.79 (t, *J* = 5.1 Hz, H-14), and two oxymethylene proton signals at $\delta_H$ 4.34 (d, *J* = 5.1 Hz, H-15). In its $^{13}$C NMR spectrum, 20 resonances (Table 2) were classified by DEPT experiments into three methyls at $\delta_C$ 11.7, 15.4, and 17.1, seven methylenes (one oxygenated), two methines, two quaternary carbons, four olefinic carbons at $\delta_C$ 150.2 (s), 142.1 (d), 137.9 (s), and 107.4 (t), a carboxyl at $\delta_C$ 182.4, and a keto carbonyl at $\delta_C$ 203.1. The above NMR spectroscopic data and the degrees of unsaturation suggested that compound **3** should be a bicyclic-labdane diterpenoid.[6,18,19] Comprehensive analysis of 2D-NMR spectrum (HMQC, $^{1}$H-$^{1}$H COSY, and HMBC spectrum) of **3** allowed to identify that compound **3** had the same planar structure as henrilabdane C [15-hydroxy-12-oxolabda-8(17),13*E*-dien-19-oic acid].[18,19] Comparison of NMR data of **3** with those of henrilabdane C exhibited the major difference was the configuration at C-4 as judged from the obvious changes of chemical shifts of the carbons around C-4. The ROESY cross-peak (Figure 2) from $\delta_H$ 1.14 (Me-19)

**Table 2.** $^1$**H and** $^{13}$**C NMR spectroscopic data of compound 3 (400 MHz;** $CD_3OD$**;** $\delta$ **in ppm)**

| pos. | $\delta_H$ ($J$ in Hz) | $\delta_C$ | pos. | $\delta_H$ ($J$ in Hz) | $\delta_C$ |
|---|---|---|---|---|---|
| 1α | 1.17 (m) | 39.5 (t) | 11a | 3.05 (dd, 16.8, 10.0) | 33.8 (t) |
| 1β | 1.68 (m) | | 11b | 2.64 (dd, 16.8, 3.2) | |
| 2α | 1.59 (m) | 19.5 (t) | 12 | | 203.1 (s) |
| 2β | 1.63 (m) | | 13 | | 137.9 (s) |
| 3α | 1.80 (m) | 38.3 (t) | 14 | 6.79 (t, 5.1) | 142.1 (d) |
| 3β | 2.12 (m) | | 15 | 4.34 (d, 5.1) | 60.1 (t) |
| 4 | | 39.3 (s) | | | |
| 5 | 2.08 (dd, 12.5, 2.8) | 50.9 (d) | 16 | 1.72 (s) | 11.7 (q) |
| 6α | 1.36 (m) | 27.4 (t) | 17 | 4.68 (s) | 107.4 (t) |
| 6β | 1.46 (m) | | | 4.32 (s) | |
| 7α | 2.32 (m) | 38.2 (t) | 18 | | 182.4 (s) |
| 7β | 1.59 (m) | | 19 | 1.14 (s) | 17.1 (q) |
| 8 | | 150.2 (s) | 20 | 0.80 (s) | 15.4 (q) |
| 9 | 2.56 (d, 9.4) | 53.4 (d) | | | |
| 10 | | 39.5 (s) | | | |

to $\delta_H$ 0.80 (Me-20) suggested that Me-19 was $\beta$-oriented. In addition, the correlations of $H_2$-15 with Me-16 in the ROESY spectrum suggested a 13*E*-configuration. Therefore, the structure of **3** was identified as 15-hydroxy-12-oxolabda-8(17),13*E*-dien-18-oic acid.

**Figure 2.** Key ROESY correlations of compounds **1–3**

## Experimental Section

**General Experimental Procedures.** Optical rotations were measured on a JASCO P-1020 digital polarimeter. UV spectra were measured using a Shimadzu UV-2401 PC spectrophotometer. IR spectra were obtained on a Bruker Tensor-27 infrared spectrophotometer with KBr pellets. ESIMS spectra were recorded on a Bruker HTC/Esquire spectrometer, HRESIMS spectrum was recorded on an API Qstar Pulsar instrument. HREIMS spectra were recorded on a Waters AutoSpec Premier P776 instrument. 1D and 2D NMR experiments were performed on Bruker AM-400 and Avance III 600 instruments with TMS as the internal standard. Chemical shifts ($\delta$) were expressed in ppm with reference to the solvent signals. Column Chromatography (CC) was performed on silica gel (200–300 mesh, Qingdao Marine Chemical Ltd., Qingdao, China), MCI-gel CHP20P (75–150 $\mu$m; Mitsubishi Chemical Co.), and Sephadex LH-20 (GE Healthcare). Semi-preparative HPLC was run on Agilent 1100 liquid chromatograph with diode array detector (DAD), Zorbax-SB-C18 column (5 $\mu$m; 25 cm × 9.4 mm i.d.). Fractions were monitored by TLC (GF254, Qingdao Marine Chemical Ltd., Qingdao, China), and spots were detected with a $UV_{254}$ lamp and by heating silica gel plates sprayed with 10% $H_2SO_4$ in ethanol.

**Plant Materal.** Whole plants of *C. elatior* were collected in November 2009 from Jinghong, Yunnan Province, China, and identified by Mr. Yu Chen of Kunming Institute of Botany, CAS. A voucher specimen (No. HY0010) has been deposited in the State Key Laboratory of Phytochemistry and Plant Resources in West China, Kunming Institute of Botany, Chinese Academy of Sciences.

**Extraction and Isolation.** The dried whole plants (5.4 kg) were powered and extracted with 95% EtOH under reflux (3 × 20 L) and filtrated. The filtrate was concentrated and partitioned between EtOAc (3 × 2.0 L) and $H_2O$. The EtOAc fraction (201 g) was chromatographed on silica gel column eluting with petroleum ether-acetone (200:1→5:1) to afford fractions I–VI. Fraction I and II were mainly fatty acids and sterols. Fraction III (12 g) was further isolated and purified by silica gel (petroleum ether-EtOAc, 150:1) to yield **4** (11 mg). Fraction IV (62 g) was subjected to an MCI gel column chromatography (MeOH-$H_2O$, 1:1→1:0, v/v) to get subfractions IVa and IVb. Fr. IVa was separated by silica gel CC (petroleum ether-EtOAc, 10:1) to get **5** (7 mg), while Fr. IVb was separated by silica gel CC (petroleum ether-EtOAc, 10:1) and then semi-preparative HPLC to obtain **1** (2 mg), **6** (3 mg), **7** (10 mg), and **8** (11 mg). Fraction V (31g) was dealt with a MCI gel and a silica gel CC ($CHCl_3$-MeOH, 200:1) to afford **11** (15 mg). Fraction VI (45 g) was subjected to a MCI gel CC to get three subfractions (VIa, VIb, and VIc), which were extensively purified on a silica gel column ($CHCl_3$-MeOH, 100:1) and Sephadex LH-20 (MeOH) to afford **9** (15 mg) and **10** (8 mg) from Fr. VIa, afford **3** (11 mg) from Fr. VIb, and afford **2** (9 mg) from Fr. VIc.

**Chlorelactone A (1):** white amorphous powder; $[\alpha]_D^{13}$ + 21.3 ($c$ 0.20, MeOH); UV (MeOH) $\lambda_{max}$ (log $\varepsilon$) 238 (2.16) nm; IR (KBr) $\nu_{max}$ 3447, 2954, 2926, 2869, 2853, 1746, 1680, 1462, 1444, 1110, 1075, 1035, 1019 $cm^{-1}$; $^1$H and $^{13}$C NMR data see Table 1; ESIMS $m/z$ 273 $[M + Na]^+$; HRESIMS, $m/z$ 273.1463 ($[M + Na]^+$, calcd. for $C_{15}H_{22}O_3Na$, 273.1466).

**Chlorelactone B (2):** white amorphous powder; $[\alpha]_D^{26}$ + 48.8 ($c$ 0.15, MeOH); UV (MeOH) $\lambda_{max}$ (log $\varepsilon$) 225 (2.22) nm; IR (KBr) $\nu_{max}$ 3433, 2926, 2858, 1751, 1660, 1437, 1382, 1333,

1292, 1265, 1120, 1104, 1069, 1050 cm$^{-1}$; $^{1}$H and $^{13}$C NMR data see Table 1; ESIMS *m/z* 285 [M + Na]$^{+}$; HREIMS, *m/z* 262.1211 ([M]$^{+}$, calcd. for $C_{15}H_{18}O_4$, 262.1205).

**Elatiorlabdane (3):** white amorphous powder; $[\alpha]^{26}_{D}$ – 18.6 (*c* 0.26, MeOH); UV (MeOH) $\lambda_{max}$ (log *ε*) 228 (2.88) nm; IR (KBr) $\nu_{max}$ 3442, 2930, 2868, 1694, 1680, 1643, 1461, 1451, 1387, 1275, 1021 cm$^{-1}$; $^{1}$H and $^{13}$C NMR data see Table 2; ESIMS *m/z* 333 [M – H]$^{-}$; HREIMS, *m/z* 334.2144 ([M]$^{+}$, calcd. for $C_{20}H_{30}O_4$, 334.2144).

## Electronic Supplementary Material

Supplementary material is available in the online version of this article at http://dx.doi.org/ 10.1007/s13659-012-0039-7 and is accessible for authorized users.

## Acknowledgments

This work was supported by National Natural Science Funding of China (No. 81072543), the Key Project of Knowledge Innovation Project of CAS (No. KSCX2-EW-Q-10), and funds (No. P2009-KF04) from the State Key Laboratory of Phytochemistry and Plant Resources in West China.

**Open Access** This article is distributed under the terms of the Creative Commons Attribution License which permits any use, distribution, and reproduction in any medium, provided the original author(s) and source are credited.

## Electronic supplementary material

Supplementary material, approximately 4.36 MB.
