# Supplementary material for: Terpenoids from Chloranthus elatior
Source: Nat Prod Bioprospect. 2012 Jul 2;2(4):156–9. doi: 10.1007/s13659-012-0039-7 (PMC4131625; doi:10.1007/s13659-012-0039-7)

Electronic Supplementary Material

Terpenoids from *Chloranthus elatior*

Chang-Li SUN,[a,b] Huan YAN,[a] Xu-Hong LI,[a] Xue-Fang ZHENG,[a] and Hai-Yang LIU[a,*]

[a]State Key Laboratory of Phytochemistry and Plant Resources in West China, Kunming Institute of Botany, Chinese Academy of Sciences, Kunming 650201, China

[b]College of Forestry, Southwest Forestry University, Kunming 650224, China

Received 21 May 2012; Accepted 25 June 2012

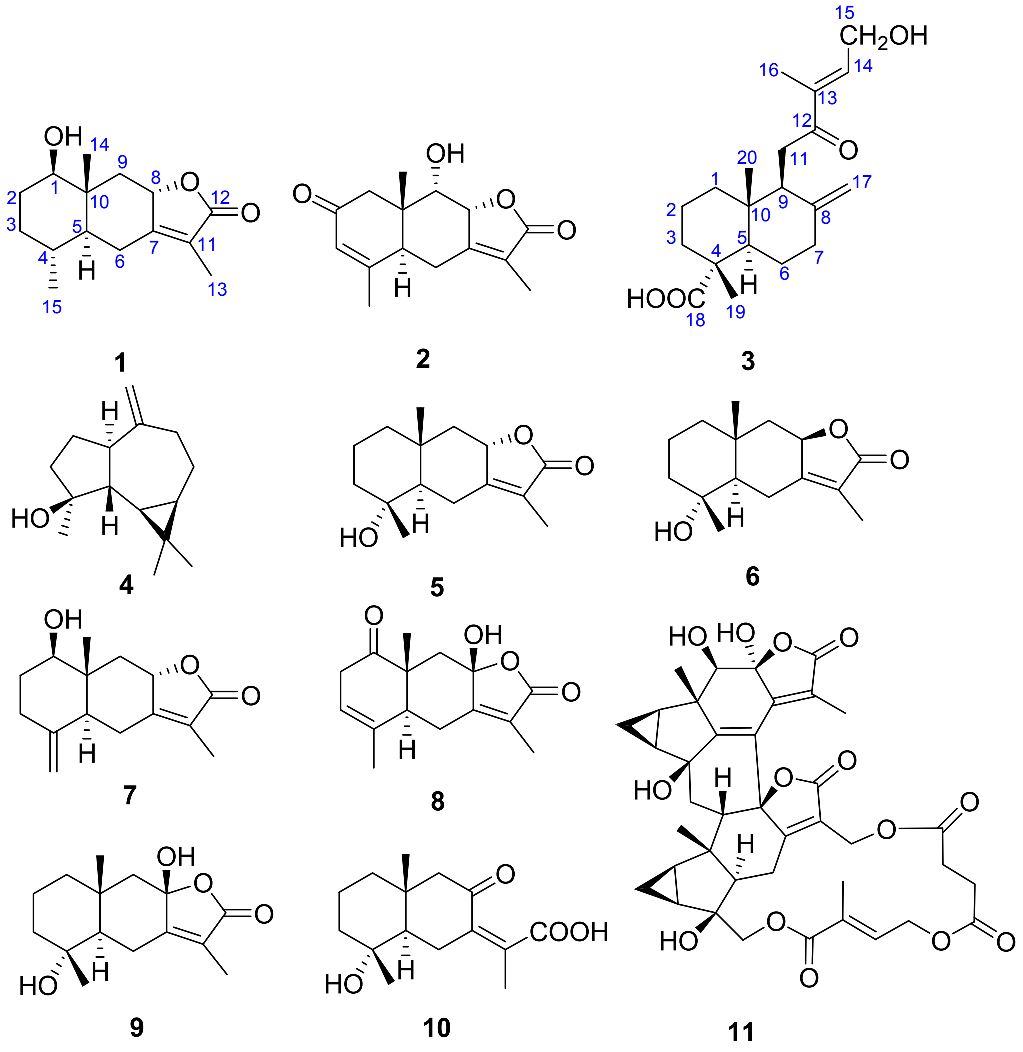

Structures of compounds **1–11**

*To whom correspondence should be addressed. E-mail: haiyangliu@mail.kib.ac.cn

Table of contents

Figure S1. ^{1}H NMR spectrum of Chlorelactone A (**1**) in $CDCl_3$.

Figure S2. ^{13}C NMR spectrum of Chlorelactone A (**1**) in $CDCl_3$.

Figure S3. HSQC spectrum of Chlorelactone A (**1**) in $CDCl_3$.

Figure S4. HMBC spectrum of Chlorelactone A (**1**) in $CDCl_3$.

Figure S5. ^{1}H-^{1}H COSY spectrum of Chlorelactone A (**1**) in $CDCl_3$.

Figure S6. ROESY spectrum of Chlorelactone A (**1**) in $CDCl_3$.

Figure S7. ^{1}H NMR spectrum of Chlorelactone B (**2**) in CD_3OD.

Figure S8. ^{13}C NMR spectrum of Chlorelactone B (**2**) in CD_3OD.

Figure S9. HSQC spectrum of Chlorelactone B (**2**) in CD_3OD.

Figure S10. HMBC spectrum of Chlorelactone B (**2**) in CD_3OD.

Figure S11. ^{1}H-^{1}H COSY spectrum of Chlorelactone B (**2**) in CD_3OD.

Figure S12. ROESY spectrum of Chlorelactone B (**2**) in CD_3OD.

Figure S13. ^{1}H NMR spectrum of Elatiorlabdane (**3**) in CD_3OD.

Figure S14. ^{13}C NMR spectrum of Elatiorlabdane (**3**) in CD_3OD.

Figure S15. HSQC spectrum of Elatiorlabdane (**3**) in CD_3OD.

Figure S16. HMBC spectrum of Elatiorlabdane (**3**) in CD_3OD.

Figure S17. ^{1}H-^{1}H COSY spectrum of Elatiorlabdane (**3**) in CD_3OD.

Figure S18. ROESY spectrum of Elatiorlabdane (**3**) in CD_3OD.

Figure S1. ^{1}H NMR spectrum of Chlorelactone A (**1**) in $CDCl_3$

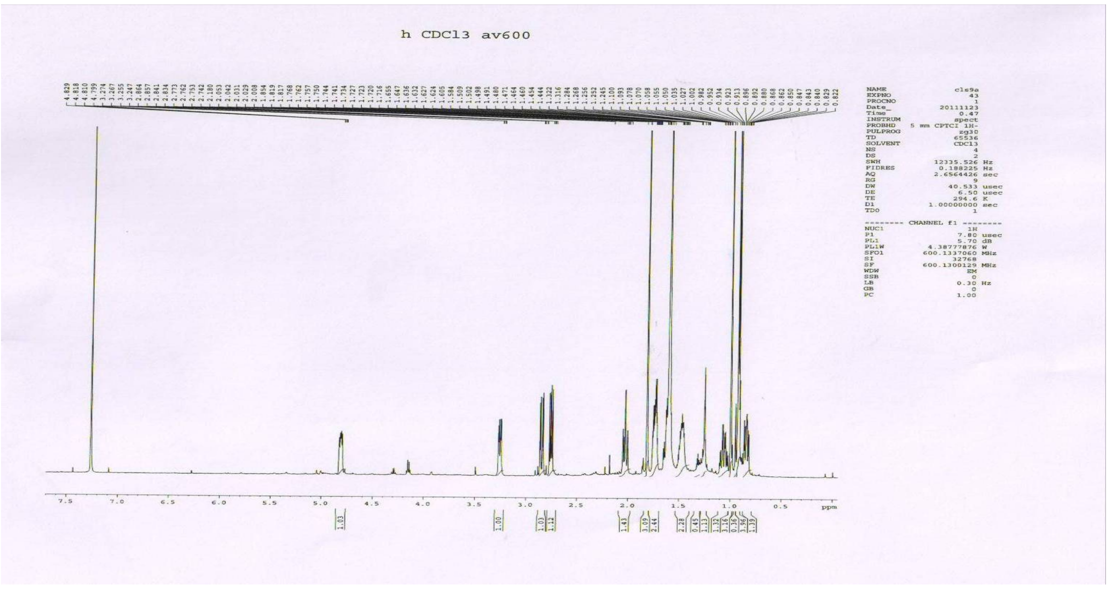

Figure S2. ^{13}C NMR spectrum of Chlorelactone A (**1**) in $CDCl_3$.

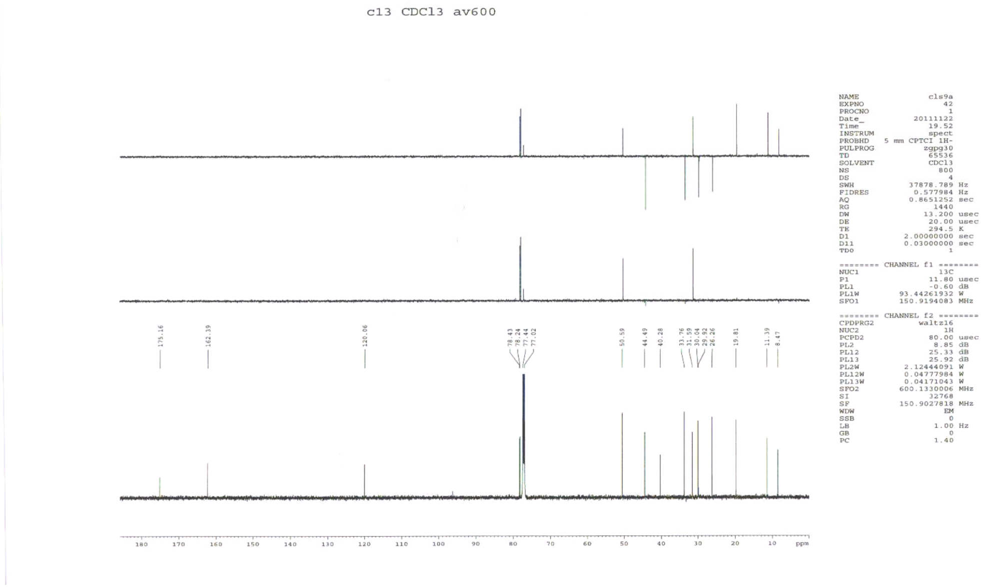

Figure S3. HSQC spectrum of Chlorelactone A (**1**) in $CDCl_3$.

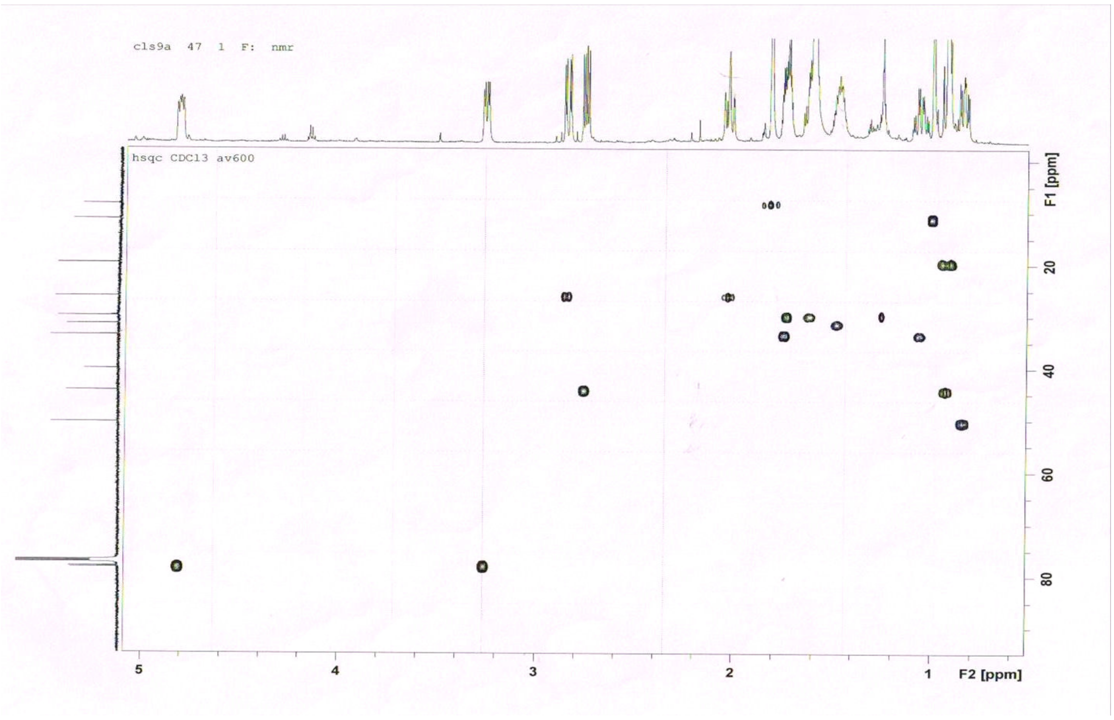

Figure S4. HMBC spectrum of Chlorelactone A (**1**) in $CDCl_3$.

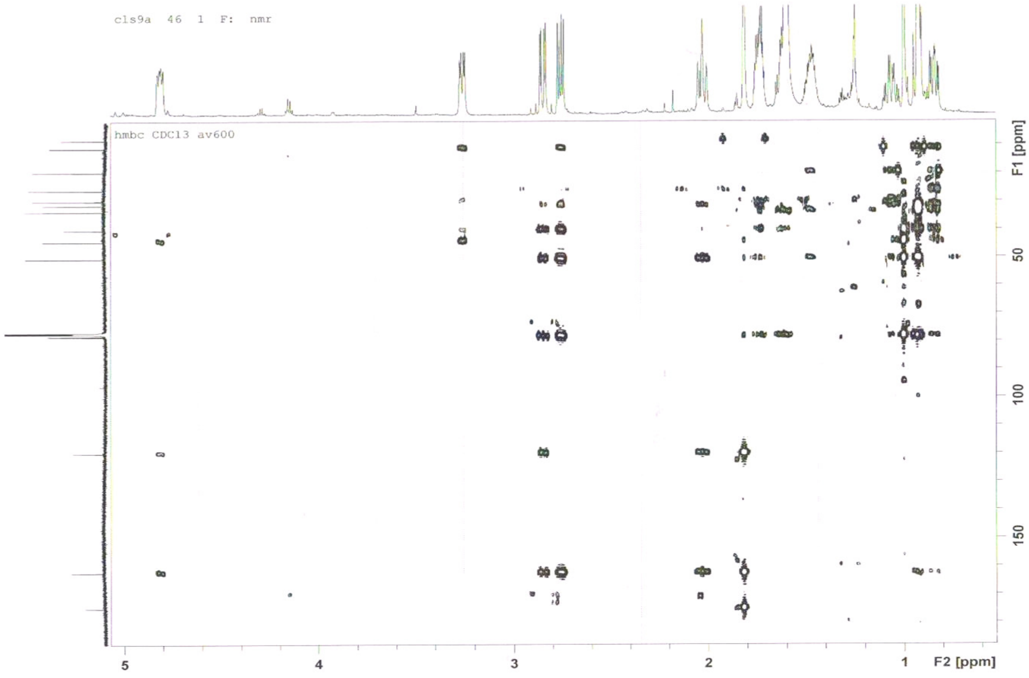

Figure S5. ^{1}H-^{1}H COSY spectrum of Chlorelactone A (**1**) in $CDCl_3$.

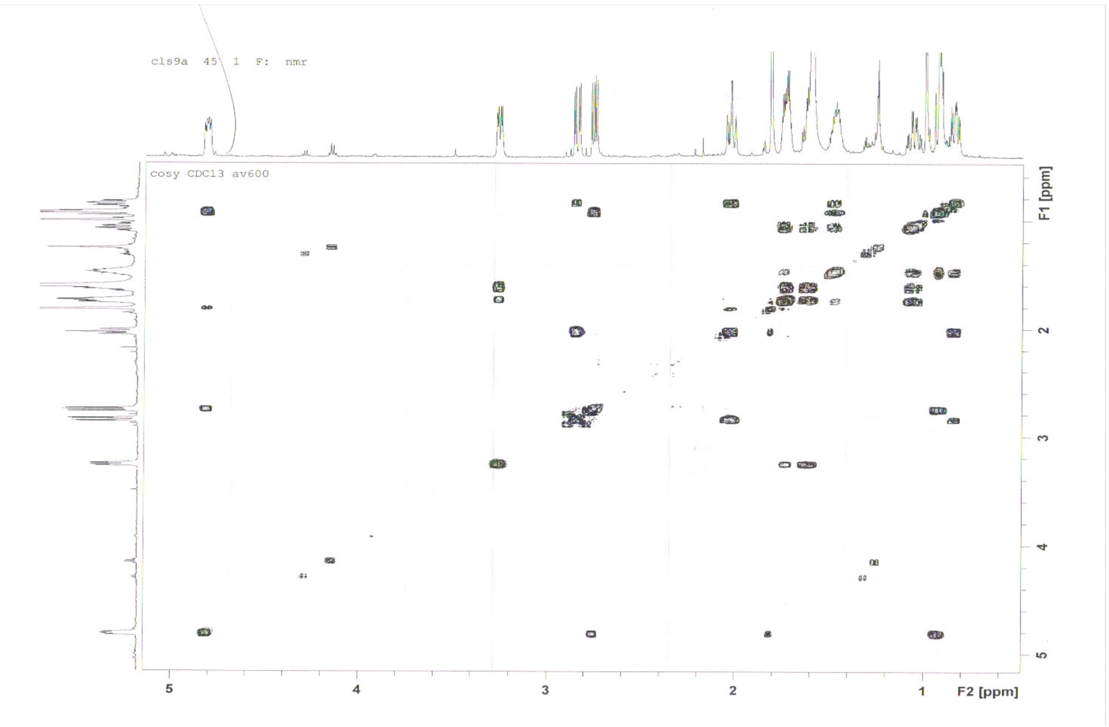

Figure S6. ROESY spectrum of Chlorelactone A (**1**) in $CDCl_3$.

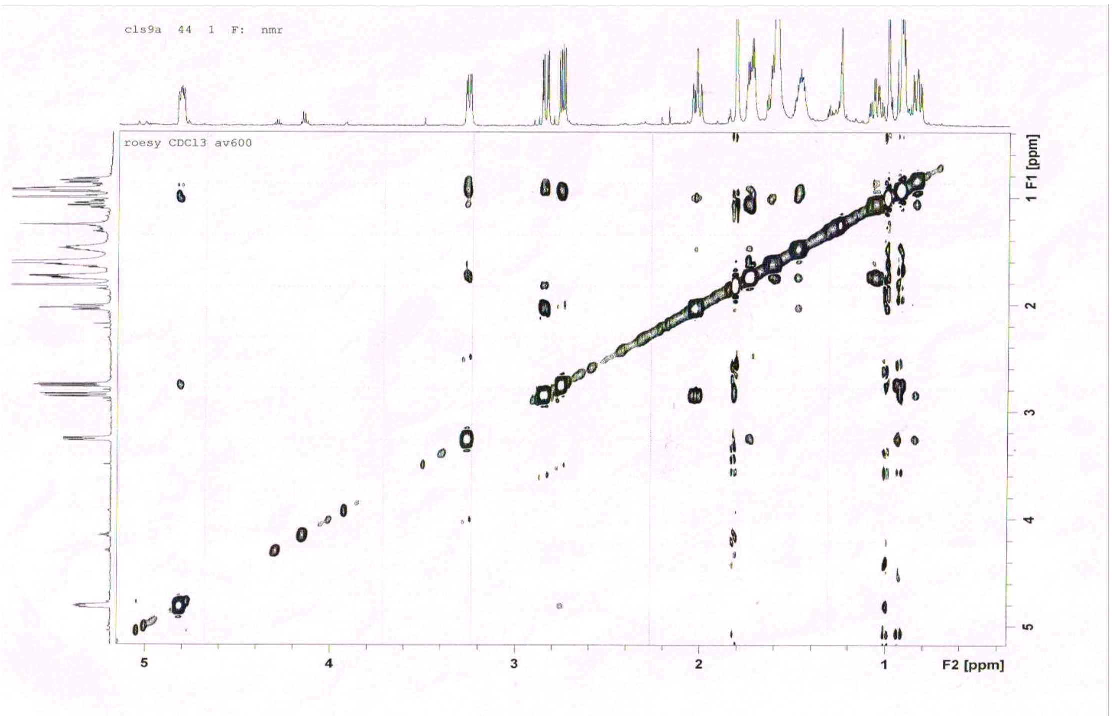

Figure S7. ^{1}H NMR spectrum of Chlorelactone B (**2**) in CD_3OD.

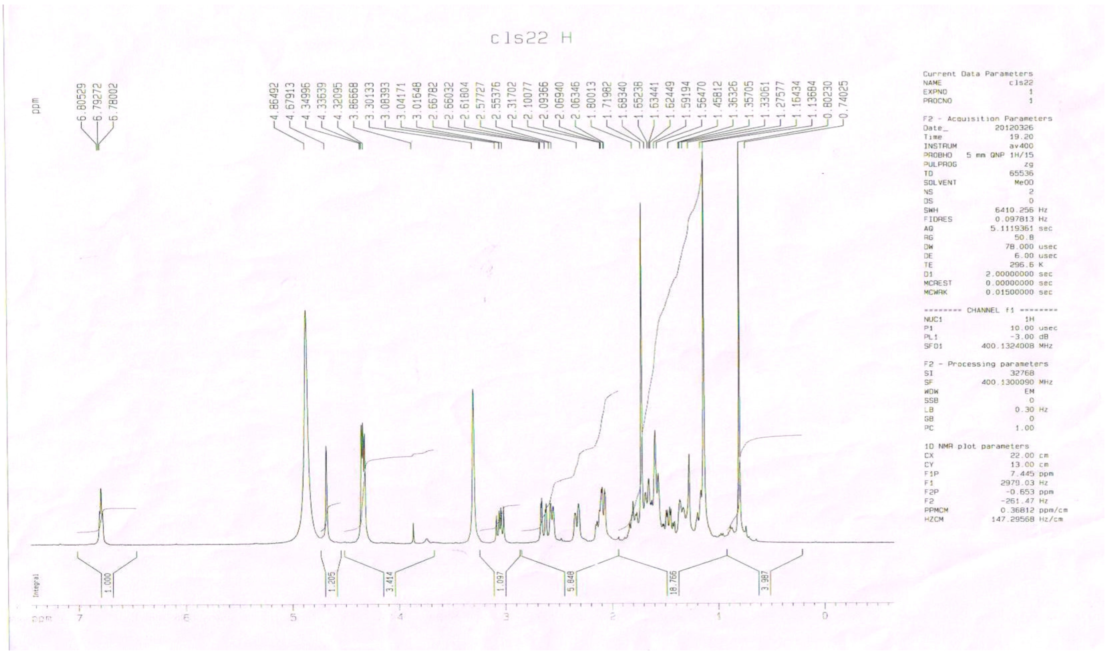

Figure S8. ^{13}C NMR spectrum of Chlorelactone B (**2**) in CD_3OD.

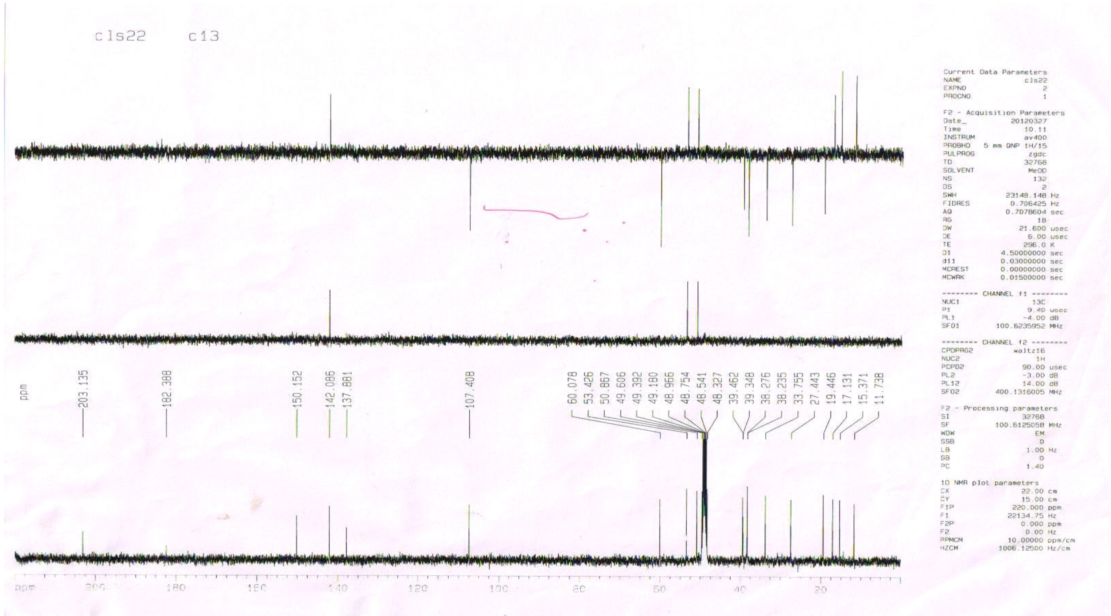

Figure S9. HSQC spectrum of Chlorelactone B (**2**) in CD_3OD.

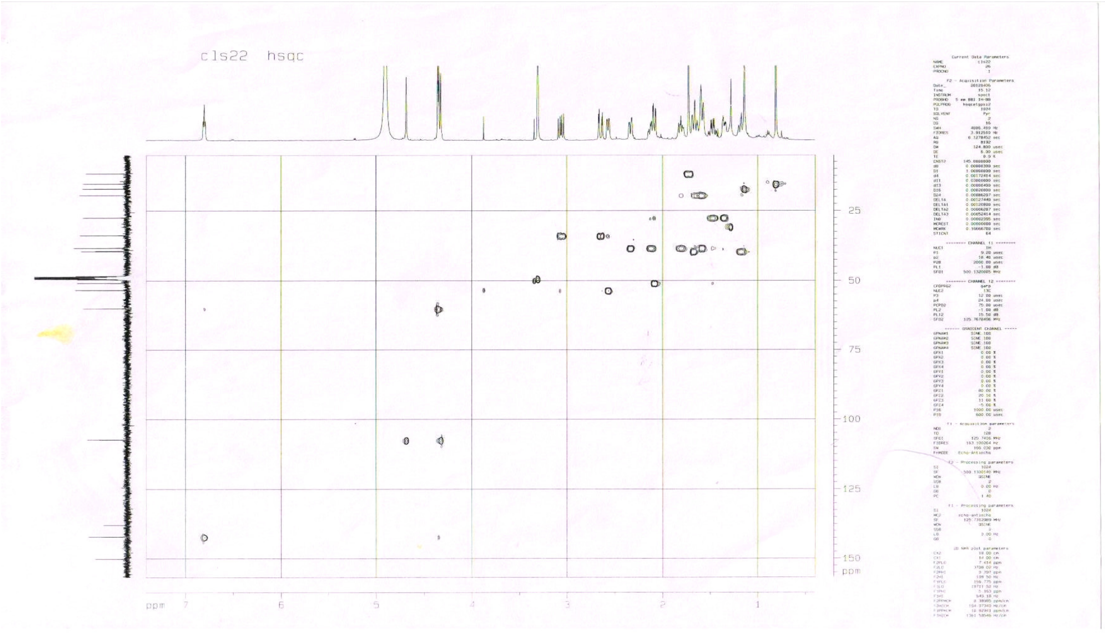

Figure S10. HMBC spectrum of Chlorelactone B (**2**) in CD_3OD.

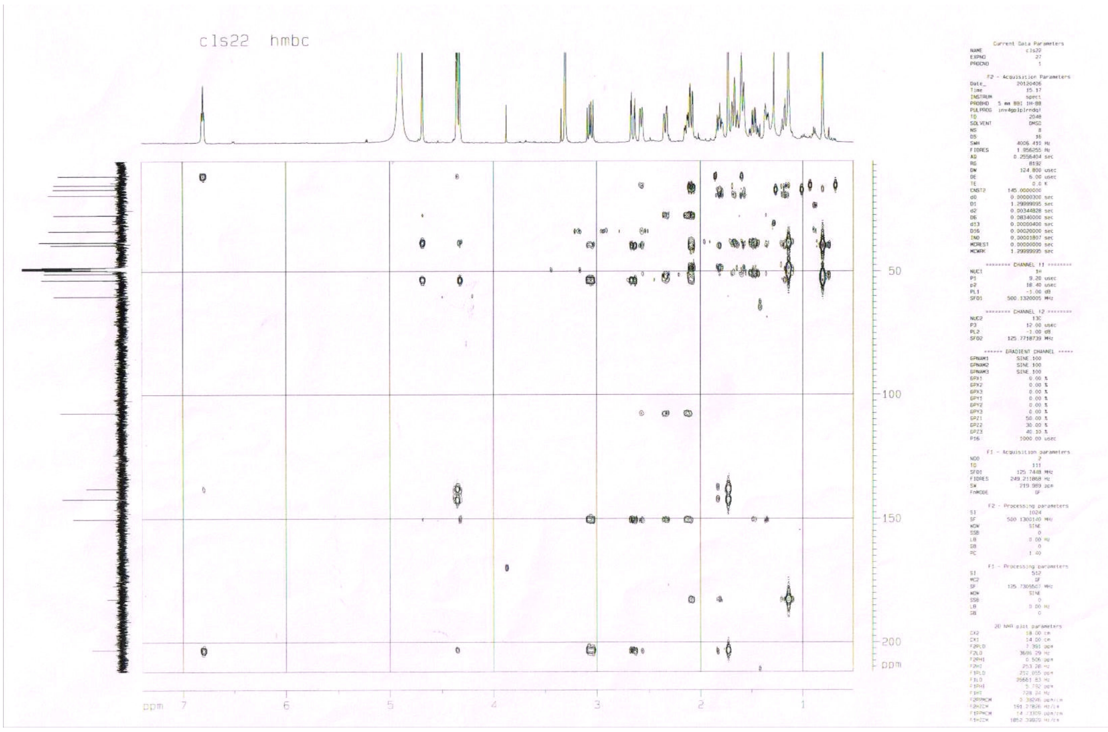

Figure S11. ^{1}H-^{1}H COSY spectrum of Chlorelactone B (**2**) in CD_3OD.

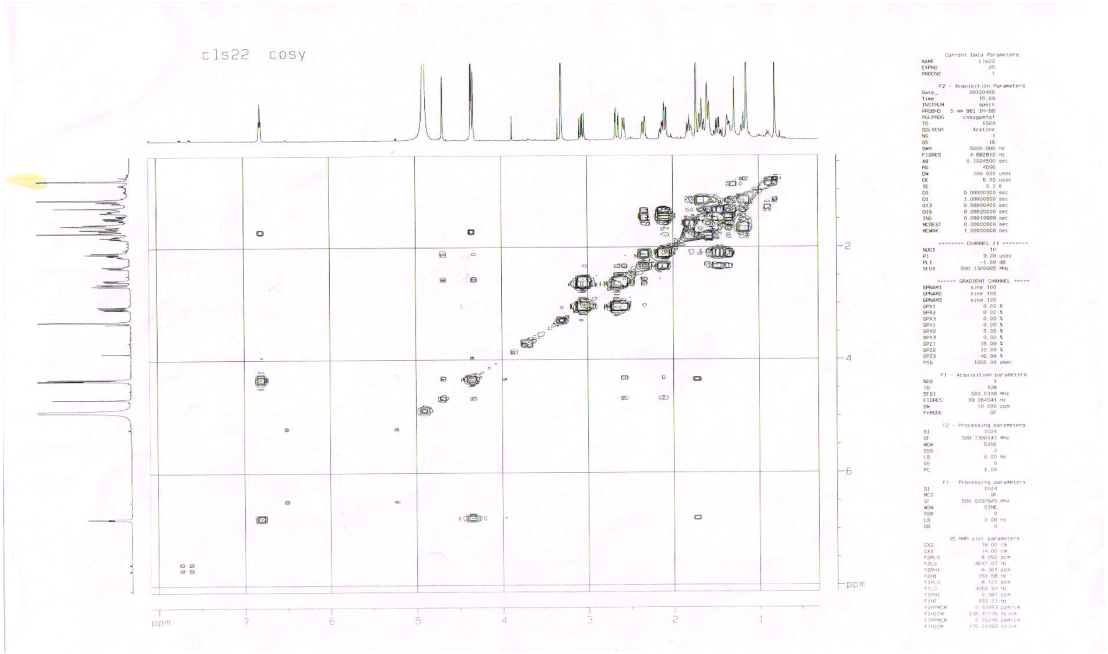

Figure S12. ROESY spectrum of Chlorelactone B (**2**) in CD_3OD.

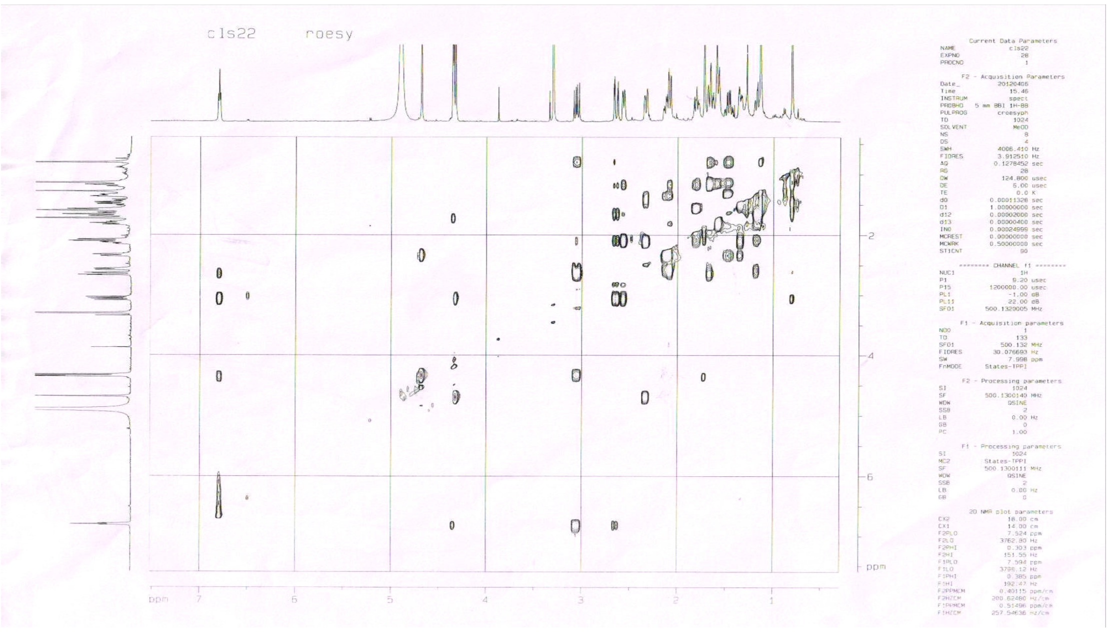

Figure S13. ^{1}H NMR spectrum of Elatiorlabdane (**3**) in CD_3OD.

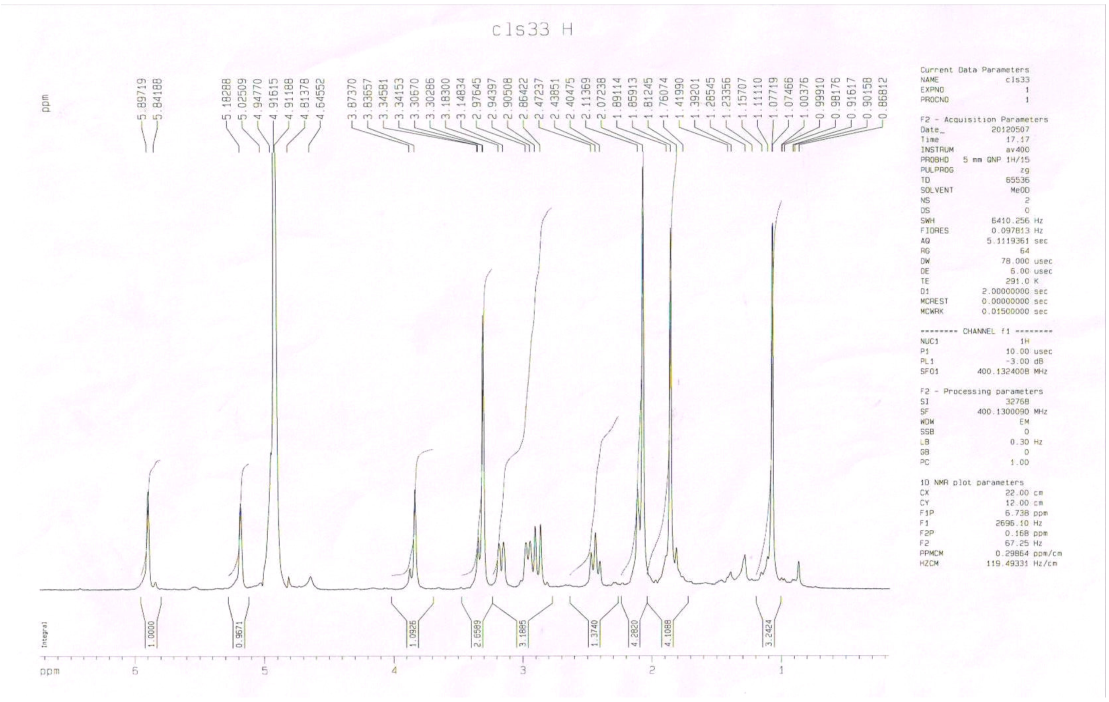

Figure S14. ^{13}C NMR spectrum of Elatiorlabdane (**3**) in CD_3OD.

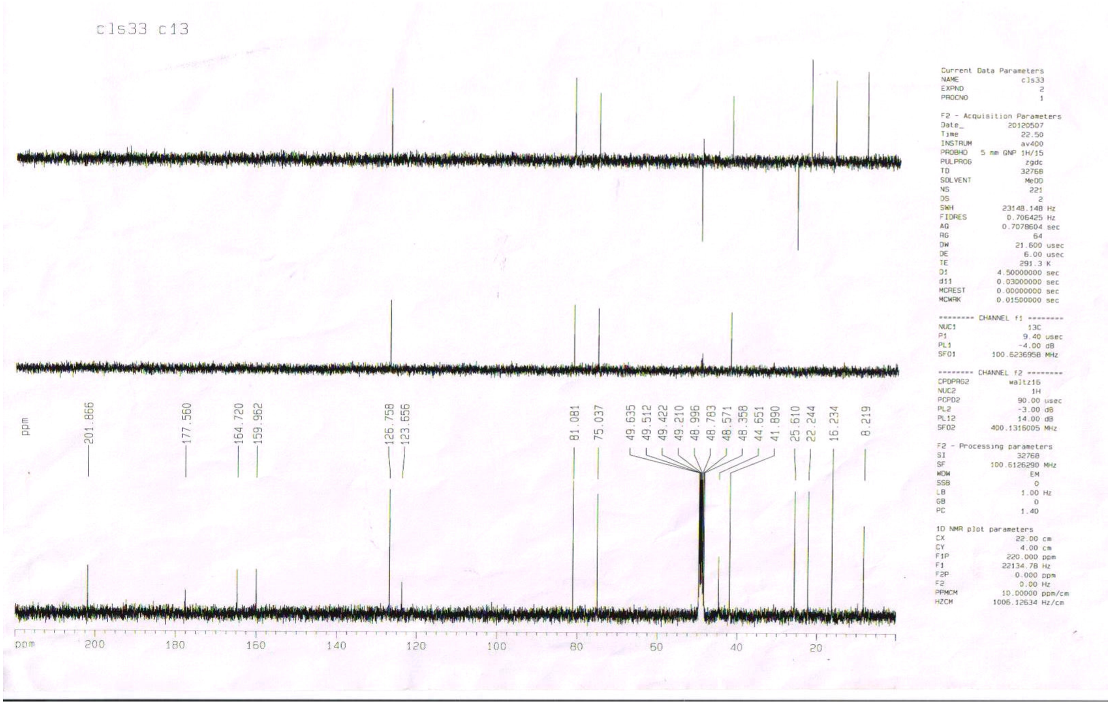

Figure S15. HSQC spectrum of Elatiorlabdane (**3**) in CD_3OD.

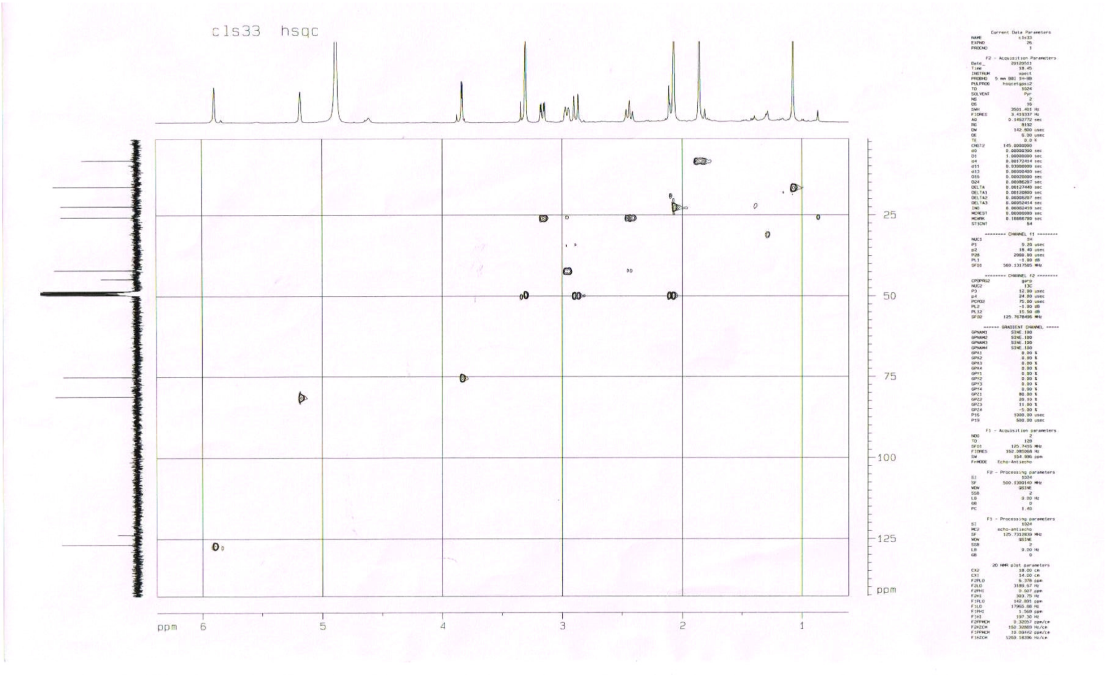

Figure S16. HMBC spectrum of Elatiorlabdane (**3**) in CD_3OD.

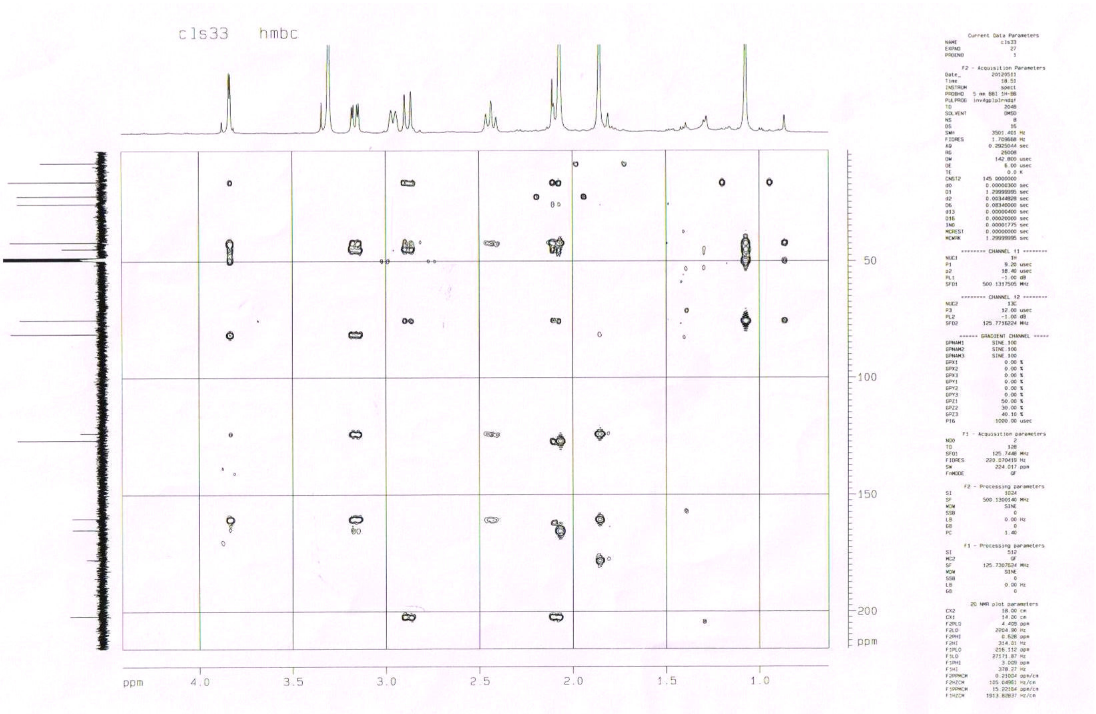

Figure S17. ^{1}H-^{1}H COSY spectrum of Elatiorlabdane (**3**) in CD_3OD.

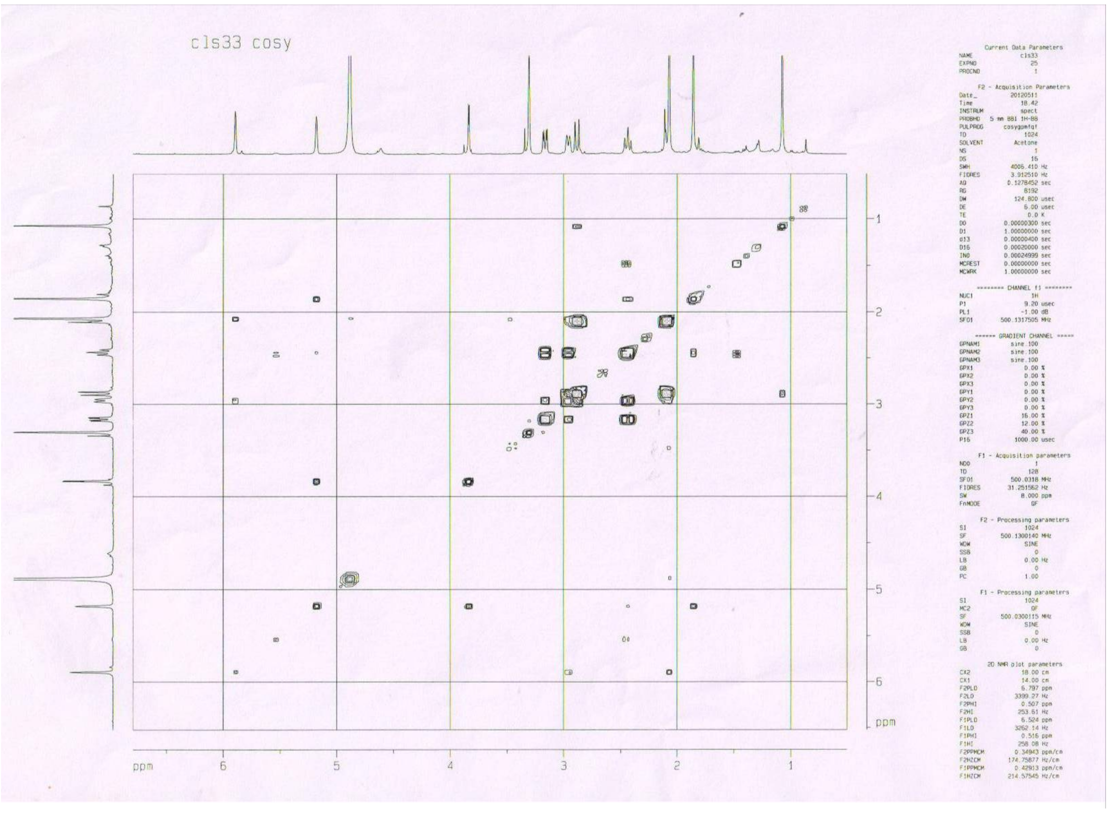

Figure S18. ROESY spectrum of Elatiorlabdane (**3**) in CD_3OD.

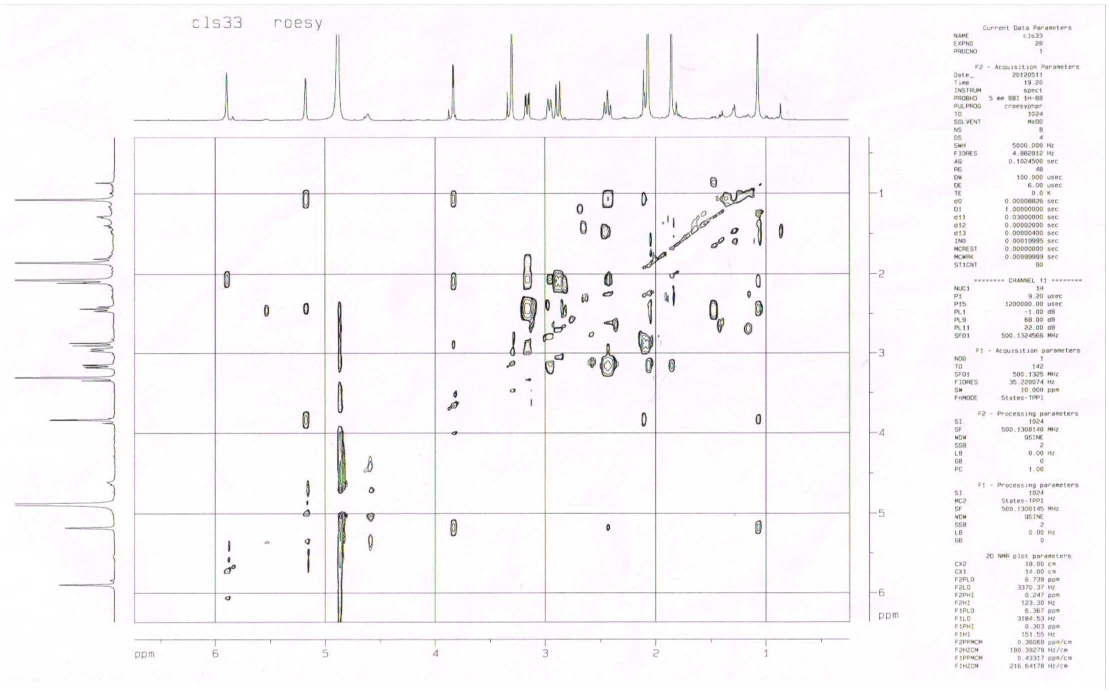

Supplement: Supplementary file 1 — Supplementary material, approximately 4.36 MB. [file 13659_2012_39_MOESM1_ESM.pdf]
